# Cutaneous Sarcoidosis Induced by Laser Therapy: Case Report and Review of the Literature

**DOI:** 10.3390/life14060773

**Published:** 2024-06-17

**Authors:** Hanna Cisoń, Magdalena Simon-Błażewicz, Joanna Suseł, Marianna Suseł, Zdzisław Woźniak, Rafał Białynicki-Birula, Jacek C. Szepietowski

**Affiliations:** 1 Department of Dermatology, Venereology and Allergology, Wroclaw Medical University, 50-367 Wroclaw, Poland; 2 Medplus Medical Doctor’s Office M. Simon-Błażewicz, 58-100 Świdnica, Poland; 3 JM Susel Clinic, 51-504 Wroclaw, Poland; 4 Department of General and Experimental Pathology, Wroclaw Medical University, 50-367 Wroclaw, Poland

**Keywords:** skin, skin pathology, sarcoidosis, sarcoidosis drug therapy, lasers, laser adverse effects

## Abstract

Background: Sarcoidosis, characterized by non-caseating epithelioid granulomas, presents diagnostic and therapeutic challenges. Method: Here we present a 38-year-old woman who exhibited erythematous and infiltrated skin lesions on her facial region following fractional laser treatment. Results: Histological analysis confirmed cutaneous sarcoidosis. Initial interventions with topical clobetasol and oral chloroquine provided transient relief. Subsequent outpatient management comprised topical tacrolimus and clobetasol, as well as systemic methotrexate, later substituted with prednisone. Gradual tapering resulted in lesion reduction. Conclusions: This case underscores the intricate nature of cutaneous sarcoidosis and the necessity for personalized therapeutic approaches. The association with cosmetic procedures highlights the importance of understanding potential triggers. The presented case highlights and reminds the medical community that lasers are not only used for therapeutic purposes but can also induce specific responses through laser therapy. Notably, while laser therapy is frequently employed in treating cutaneous sarcoidosis, its role in inducing sarcoidosis warrants further investigation.

## 1. Introduction

Sarcoidosis is a multifaceted systemic condition typified by granuloma formation, characteristically non-caseating epithelioid granulomas, in diverse organ systems. Scientific inquiry has illuminated the interplay of genetic susceptibility and environmental elements as key contributors to the etiology of an augmented immune response, culminating in the development of sarcoidosis [1]. The lungs and thoracic lymph nodes are the predominant sites of involvement, observed in over 90% of cases. Nevertheless, it is important to note that nearly every other organ system can potentially exhibit manifestations of the condition [2]. Dermatological involvement is a prevalent occurrence, with documented incidence rates exhibiting a range between 9% and 37% [3,4,5].

Sarcoidosis is a disease that affects individuals across all age groups, independent of their racial or ethnic background. However, it is most frequently diagnosed in individuals aged between 20 and 39 years [1]. It is noteworthy that this condition exhibits a higher prevalence among female individuals, non-smokers, and those residing in rural environments [6]. In the European context, there is a distinct geographic variation in the incidence of the disease, with northern regions reporting a considerably higher occurrence, estimated at approximately 60 cases per 100,000 individuals, in contrast to southern European countries like Italy, where the incidence is notably lower, at less than 10 cases per 100,000 individuals [7]. Exposure to a range of substances, including wood stove emissions, soil components, tree pollen, inorganic particulates, insecticides, and silica, has been linked to an increased susceptibility to sarcoidosis development [8,9].

Furthermore, it is postulated that certain microorganisms, such as *Mycoplasma* species, *Leptospira* species, *Herpesviruses*, *Retroviruses*, *Chlamydia pneumoniae*, *Borrelia burgdorferi*, *Pneumocystis jirovecii*, and *Propionibacterium* species, may be a potential triggers of sarcoidosis [10,11,12]. Furthermore, individuals subjected to interferon α therapy for the treatment of hepatitis C infection have been reported to manifest sarcoidosis as a notable consequence [13]. Moreover, drug-induced sarcoidosis-like reactions have been documented in connection with various pharmaceutical agents, including checkpoint inhibitors, anti-tumor necrosis factor (TNF), and anti-interleukin biological agents [14].

Here, we present the case of a 38-year-old female patient presenting with erythematous and infiltrated skin lesions on her facial region. These cutaneous manifestations became apparent approximately one month after ablative fractional CO_2_ laser treatment, as confirmed by histological examination revealing cutaneous sarcoidosis. Our patient represents the third reported case of laser-induced cutaneous sarcoidosis in the currently available literature. The literature search was conducted in the PubMed and Google Scholar databases, using combinations of the following relevant keywords: cutaneous sarcoidosis, laser therapy, and laser. Written informed consent for use of the patient’s data for this study, histopathologic preparation of the tissues, and publication of the relevant data was obtained from the family of our deceased patient.

## 2. Clinical Case Presentation

**Figure 1:**
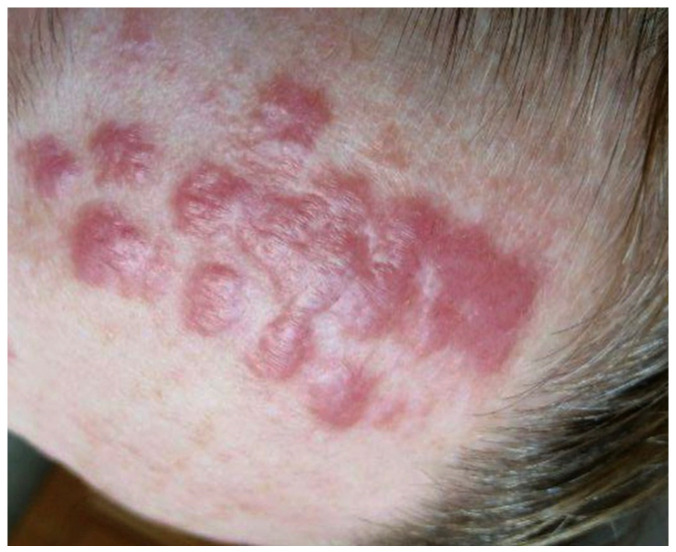
The patient’s forehead with the numerous erythematous, infiltrated skin lesions.

**Figure 2:**
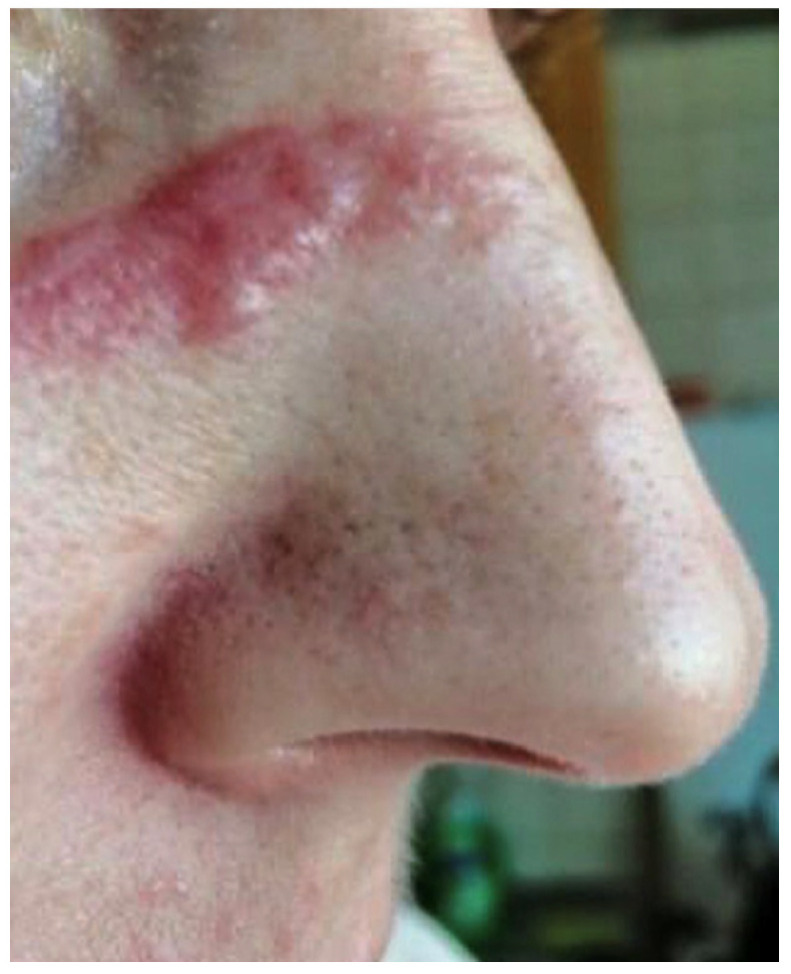
The erythematous, infiltrated skin lesions on the nose of the patient.

A 38-year-old female patient was admitted to the Department of Dermatology, Venereology and Allergology in Wrocław, Poland, due to the presence of erythematous and infiltrated skin lesions on her facial region (Figure 1 and Figure 2).

Cutaneous manifestations manifested approximately one month post ablative fractional CO_2_ laser (wavelength of 10,600 nm and a fluence of 5 J/cm^2^ with a pulse duration of <1 ms) treatment. The laser therapy, conducted by a non-medical aesthetician, utilized a 125 mm handpiece, an 8 mm spot size, 25% density, and 12 Watts. The procedure consisted only of one session.

It is also noteworthy that in the patient’s medical history there was information about a 7-month course of isotretinoin therapy for acne four weeks prior to the laser procedure.

Isotretinoin was administered orally to the patient at a dosage of 20 mg twice daily, demonstrating favorable tolerability and maintaining results within normal parameters. Notably, complete remission of pathological manifestations was achieved during the final 3 months of isotretinoin therapy.

**Figure 3:**
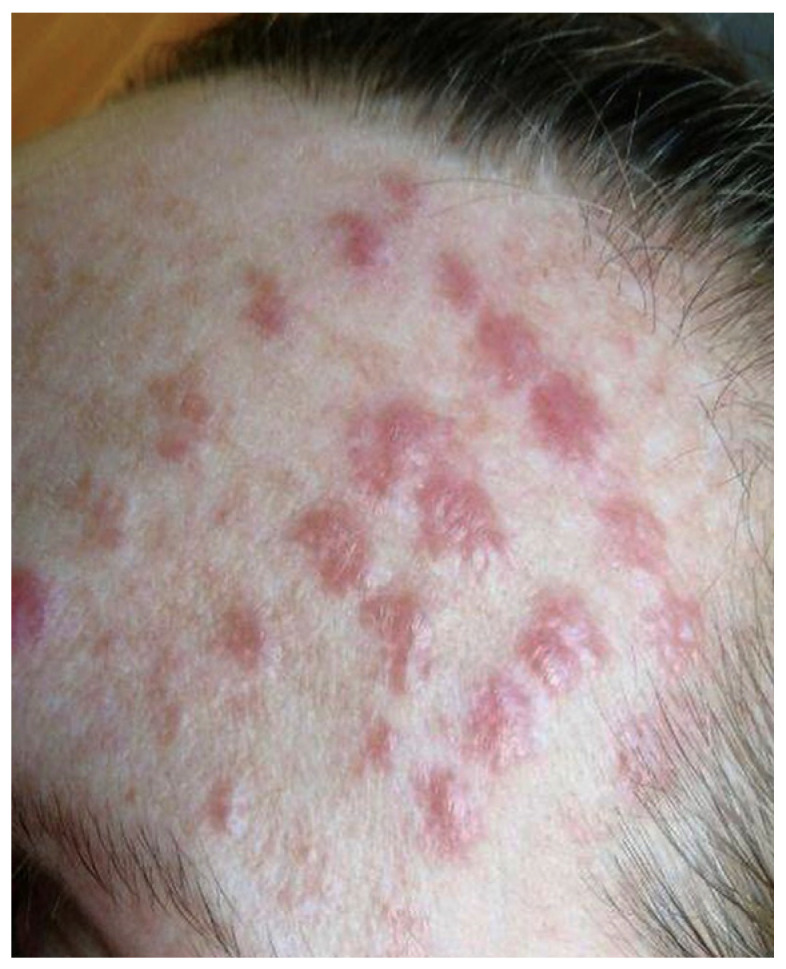
The recurrence of the intensity of infiltration on the left forehead.

**Figure 4:**
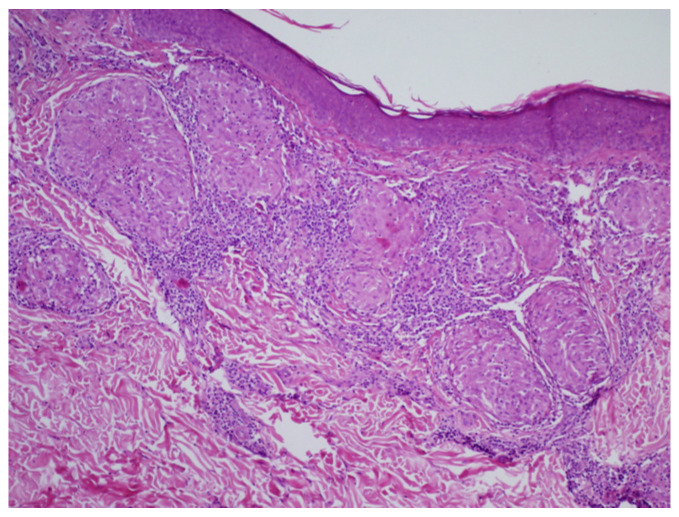
The histological features confirmed cutaneous sarcoidosis (H&E staining, 100×).

Initially, in the outpatient department, oral acyclovir was prescribed, suspecting herpes virus etiology for the skin lesions; however, this treatment failed to yield any improvement. Subsequently, a therapeutic regimen involving topical clobetasol propionate 0.5 mg/g and oral chloroquine at a dose of 250 mg b.i.d. for 2 months was initiated, due to suspicion of cutaneous sarcoidosis, leading to a reduction in erythema and infiltration. Regrettably, upon discontinuation of this treatment, the skin lesions recurred (Figure 3). Upon the resumption of topical clobetasol, no significant improvement was observed. In the course of outpatient diagnostics, a skin biopsy was performed. The histological examination revealed granulomatous inflammation mainly in superficial dermis without involvement of the subcutis. The confluent granulomata were composed of epithelioid histiocytes with abundant eosinophilic cytoplasm and oval vesicular nuclei. Few multinucleated giant cells were present. The granulomas were surrounded with few surrounding lymphocytes (‘naked granulomas’) and a rim of dermal fibrosis. Discrete small central foci of fibrinoid necrosis were present. The epidermis displayed mild acanthosis. The histological features confirmed cutaneous sarcoidosis (Figure 4).

Six months after the first hospitalization, the patient was again admitted to our department, where a thorough diagnostic workup was conducted, encompassing chest X-rays focused on lung hilum, abdominal ultrasound, and peripheral lymph node ultrasound. These investigations did not reveal any significant abnormalities. Following her hospital discharge, the patient continued her treatment regimen as an outpatient, consisting of topical tacrolimus (0.1% ointment) and clobetasol (cream 0.05%), resulting in a modest improvement.

Two months later, during her next hospitalization due to cutaneous sarcoidosis, erythematous and infiltrated skin changes were once again observed, primarily on her forehead, cheeks, and nose, albeit with a lesser degree of severity compared to her prior hospitalization. Laboratory tests performed at that time revealed results within the normal range, and the Quantiferon test was negative. Consequently, a decision was made to initiate methotrexate therapy at a dosage of 15 mg per week administered s.c., and this treatment was continued as part of her management plan. The methotrexate was changed to prednisone due to unsatisfactory treatment outcomes.

**Figure 5:**
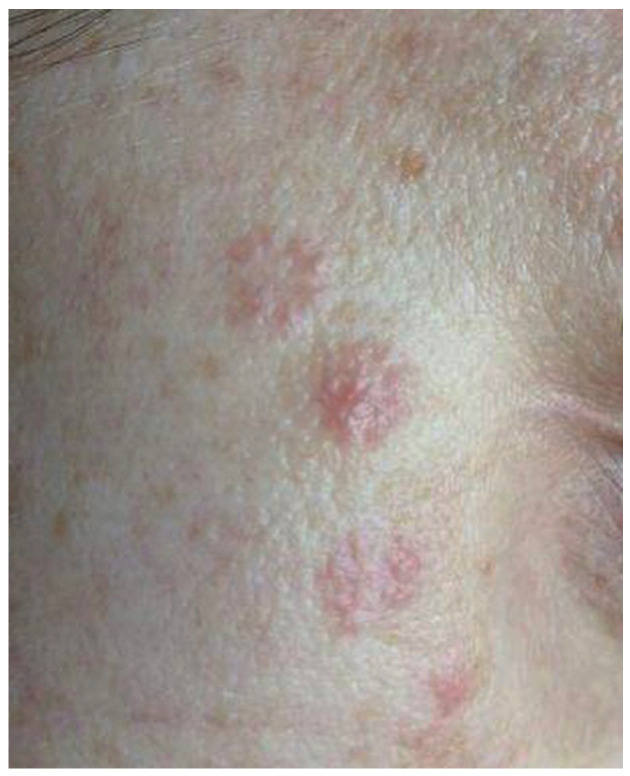
The healing due to prednisone therapy—the less infiltrated skin lesions on the right cheek.

In conclusion, the gradual tapering of prednisone doses (20-10-5 mg p.d.) demonstrated favorable outcomes, leading to a reduction in cutaneous manifestations (Figure 5).

## 3. Discussion and Review of the Literature

### 3.1. Symptoms of Cutaneous Sarcoidosis

Cutaneous manifestations of sarcoidosis can be categorized into two distinct classes: specific and nonspecific lesions [3].

Specific lesions are characterized by histological confirmation of typical sarcoid granulomas and are often chronic and asymptomatic, requiring therapeutic intervention. These lesions include macules, papules, plaques, lupus pernio, and subcutaneous nodules, known as Darier–Roussy nodules [3,5,15]. Less common variations consist of skin changes associated with scars and tattoos, as well as hypopigmented, angiolupoid, psoriasiform, erythrodermic, ulcerative, ichthyosiform, verrucous, and lichenoid forms [3,15,16].

Nonspecific lesions, on the other hand, result from an inflammatory reaction pattern. Erythema nodosum, a frequently observed nonspecific skin manifestation, is often associated with acute sarcoidosis and is typically indicative of a favorable prognosis [17]. Understanding and distinguishing these cutaneous presentations is essential for proper diagnosis and management of sarcoidosis.

### 3.2. Diagnosis of Cutaneous Sarcoidosis

When there is a clinical suspicion of sarcoidosis, the performance of a skin biopsy is imperative. In our study, histological examination was typical and revealed granulomatous inflammation in superficial dermis. Confluent granulomata with few multinucleated giant cells were surrounded with minimal lymphocytic infiltration and a rim of fibrosis. However, discrete small central foci of fibrinoid necrosis were present.

Subsequent to the confirmation of cutaneous sarcoidosis through biopsy, it is essential to undertake a comprehensive evaluation to assess the potential for systemic involvement [18].

### 3.3. Treatment of Cutaneous Sarcoidosis

Topical treatments are preferred, if possible, especially for cutaneous or bronchial sarcoidosis. Severe, persisting cases require long-term immunosuppressive drugs, with glucocorticoids as the primary choice [19]. Methotrexate or rarely azathioprine, mycophenolate mofetil, and immunomodulators like hydroxychloroquine are alternative options. Tumor necrosis factor-alpha inhibitors, such as adalimumab and infliximab, are reserved as third-line agents but may be considered earlier for severe cases, organ involvement, or intolerance to standard drugs [18].

### 3.4. Cosmetic Procedure and Cutaneous Sarcoidosis

In the contemporary scientific literature, there exist documented instances of cutaneous sarcoidosis presenting in locations where previous blepharoplasty, tattooing, skin piercing, injections, or permanent makeup procedures, such as micro-blading, have been performed [5,20,21,22].

### 3.5. Laser Therapy and Cutaneous Sarcoidosis

While the existing literature discusses the treatment of cutaneous sarcoidosis with laser therapy [23,24,25,26,27,28,29,30,31,32,33,34,35] (Table 1), there are also singular occurrences describing laser therapy causing cutaneous sarcoidosis [36,37] (Table 2).

#### 3.5.1. Pulsed Dye Laser

The Pulsed Dye Laser (PDL) was designed with the objective of implementing selective thermolysis for vascular lesions, with hemoglobin serving as the principal chromophore.

In a study by Roos et al. [24], a 63-year-old Caucasian woman presented with well-defined nodules on her back, emerging over a 2-week period. Histological examination confirmed cutaneous sarcoidosis. Fractionated Pulsed Dye Laser (FPDL) treatment resulted in complete clearance at treated sites after 4 weeks, contrasting with untreated sites.

Dong et al. [25] reported a 46-year-old Asian woman with longstanding left facial lesions, diagnosed with cutaneous sarcoidosis. Treatment included acitretin (10 mg q.d.), hydroxychloroquine (200 mg b.i.d.), and PDL therapy (595 nm, 6 ms, 7 mm, 14 J/cm^2^). Improvement was observed after 10 PDL sessions over 15 months.

Hollzman et al. [26] described a 10-year-old boy with a solitary, inflammatory, erythematous, ulcerated plaque, diagnosed with cutaneous sarcoidosis. PDL treatment (595 nm, 0.5 ms, 7 mm, 7.6–7.8 J/cm^2^) in three sessions at 6-week intervals led to favorable outcomes.

Also, Goodman et al. [27] presented a 39-year-old woman with cutaneous sarcoidosis and stable pulmonary health. Flashlamp PDL treatment (585 nm, 460 ms, 5 mm, 8 J/cm^2^) effectively reduced erythema, with optimal improvement at 8 J/cm^2^.

#### 3.5.2. Carbon Dioxide Laser

The CO_2_ laser is an ablative modality that targets water as a chromophore.

In Zaouak et al.’s study [28], a 49-year-old woman with histologically confirmed cutaneous sarcoidosis underwent experimental treatment with ablative fractional CO_2_ laser. Three sessions were administered with a 2-month interval between each, yielding satisfactory results.

O’Donoghue et al. [29] reported CO_2_ laser treatment for lupus pernio nodular component recontouring in three patients. One patient required post-treatment intralesional triamcinolone and oral prednisolone due to systemic involvement but remained recurrence-free over 6 years. Another patient maintained a disease-free status for 14 months with laser treatment alone. The third patient, receiving laser treatment with intralesional steroids, experienced partial recurrence after nine months, with improvement lasting from nine months to six years.

In Stack et al.’s study [30], a 31-year-old man with cutaneous sarcoidosis was treated with a CO_2_ laser, followed by injection of a steroid preparation into the excised regions, resulting in satisfactory outcomes.

#### 3.5.3. Intense Pulse Light

IPL employs a flashlamp to release polychromatic light spanning a wide wavelength spectrum, typically ranging from approximately 400 to 1400 nm.

Piccolo et al. [31] treated a 26-year-old woman with three nodules on her pinna, diagnosed as sarcoidosis after unsuccessful intralesional corticosteroid therapy. Following four IPL sessions (500 nm, 2 pulses of 5–10 ms, 12–16 J/cm^2^), there was a significant reduction in the vascular component and lesion consistency, along with alleviated pain.

#### 3.5.4. Combined Laser Therapies

Within the existing literature, instances emerge detailing the therapeutic approach to cutaneous sarcoidosis through the amalgamation of diverse laser modalities [32,33,34,35].

In the study by Emer J et al. [32], correction of lupus pernio on the nose was performed using PDL 545 nm and nonablative fractional resurfacing (NAFR, 1550 nm). Following the initial application of PDL (7 mm, 0.45 ms, 8 J/cm^2^), NAFR was subsequently conducted (70 mJ, treatment level 6, 8 passes). The patient expressed satisfaction with the outcomes and declined any suggested further treatments.

In investigations by Grema H et al. [33] and Ekbäck et al. [34], treatment of cutaneous sarcoidosis with PDL yielded unsatisfactory outcomes. But further treatment with a Q-switched ruby laser (10 J/cm^2^) [33] and a frequency-doubled YAG laser (532 nm, 50 ms, 12–16 J/cm^2^) [34] led to success.

Momen et al. [35] reported on several cases of cutaneous sarcoidosis treated with lasers. One patient, unresponsive to systemic treatments, saw a 50–60% improvement in upper lip erythema and plaque depth after CO_2_ and PDL sessions. Three other patients received laser treatments for lupus pernio on the nose, an erythematous forehead plaque (PDL), and cheek nodules/plaques (CO_2_ laser).

### 3.6. Laser as a Factor Inducing Cutaneous Sarcoidosis

In our investigation, the temporal proximity between the laser therapy intervention in the patient and the emergence of cutaneous sarcoidosis suggests a potential association beyond mere coincidence. The existing literature presents only two instances documenting the induction of cutaneous sarcoidosis following laser therapy [36,37].

In the investigation conducted by Kim Hr et al. [36], an observational case is detailed featuring a 71-year-old male subject with a facial scarring history spanning five decades. The individual reported a progressive enlargement of the scar subsequent to three sessions of PDL treatment administered at a dermatology clinic three years prior. Histological examination identified numerous non-caseating granulomas indicative of cutaneous sarcoidosis.

In the study by Kormeilii T. et al. [37], a clinical case was delineated involving a 33-year-old Caucasian male who initially presented with hypertrophic scars on his left glabellar region and left upper lip, resulting from a past automobile accident. The patient underwent CO_2_ laser resurfacing to ameliorate the visibility of facial scars. However, a progression was noted, with an enlargement observed in the scars on his forehead two years post-treatment. Subsequent histopathological analysis was consistent with a diagnosis of cutaneous sarcoidosis.

### 3.7. Isotretinoin and Cutaneous Sarcoidosis

In our investigation, the patient was orally administered isotretinoin for common acne prior to the laser therapy procedure. However, no description was found regarding the induction of cutaneous sarcoidosis by isotretinoin therapy. Conversely, reports exist detailing the resolution of cutaneous lesions in a patient diagnosed with cutaneous sarcoidosis following isotretinoin therapy [38,39].

## 4. Conclusions

In summary, cutaneous sarcoidosis manifests a spectrum of specific and nonspecific lesions, indicative of the intricate nature of its clinical expressions. Specific lesions, characterized by typical sarcoid granulomas, encompass diverse forms such as macules, papules, plaques, lupus pernio, and Darier–Roussy nodules. Nonspecific lesions, arising from an inflammatory reaction pattern, include erythema nodosum. The accurate diagnosis and effective management of cutaneous sarcoidosis necessitate a comprehensive understanding of these diverse cutaneous presentations.

In the literature, cutaneous sarcoidosis has been linked to various cosmetic procedures, and the role of laser therapy in both the treatment and, in rare instances, the induction of cutaneous sarcoidosis has been systematically documented. Our review accentuates distinct cases wherein laser therapy, encompassing Pulsed Dye Laser and CO_2_ laser modalities, exhibited varied efficacy in managing cutaneous sarcoidosis. Particularly noteworthy is the induction of remission by laser therapy in specific cases, underscoring its potential as a therapeutic avenue for cutaneous sarcoidosis. Our patient represents the third reported case of laser-induced cutaneous sarcoidosis in the existing literature.

Moreover, the presented cases underscore the imperative of a tailored therapeutic approach and emphasize the ongoing necessity for research endeavors aimed at elucidating optimal treatment modalities. In the context of cutaneous sarcoidosis post-laser therapy, meticulous consideration of individual patient characteristics and treatment responses is paramount. These documented cases contribute significantly to the evolving understanding of cutaneous sarcoidosis and its management, providing valuable insights into the intricate interplay between therapeutic interventions and ensuing clinical outcomes.

## Figures and Tables

**Table 1:** The use of lasers in the treatment of cutaneous sarcoidosis.

Authors and Type of the Study	Clinical Picture	Patient Age/Gender	Laser/Setting	Treatment Number	Outcome	Follow Up
Roos et al. case report [24]	Nodules on back	63 F	FPDL 585 nm 0.5 ms, 12 mm, 6 J/cm^2^	1	After four weeks, lesions had completely resolved	Prednisolone added at 4 weeks for systemic disease; at 13 months no recurrence of lesions
Dong et al. case report [25]	Erythematous papules on the left part of the face	46 F	PDL 595 nm 6 ms, 7 mm, 14 J/cm^2^	10 treatments during 15 months	The plaque was thinner, normal skin had appeared; telangiectasia was less obvious	Acitretin (10 mg per day) and hydroxychloroquine (200 mg twice a day) along with PDL therapy
Holzmann et al. case report [26]	Erythematous papule on varicella scar site on cheeks	10 M	PDL 595 mm, 0.5 ms, 7 mm, 7.6–7.8 J/cm^2^	3 treatments at 6-week intervals	Clinical remission after three treatments	No recurrence at 12 months
Goodman et al. case report [27]	Lupus pernio granulomatous papules on nasal ala	39 F	PDL 585 nm, 460 ms, 5 mm, 8 J/cm^2^	2 treatments 7 months apart	Erythema and papular components showed a 75% improvement from baseline after six months	Papules recurred at 2 months, and erythema at 6 months. By 15 months, a significant reappearance of both erythema and papules necessitated a third treatment
Zaouak et al. case report [28]	Two infiltrated papulo-nodular plaques on the cheek	49 F	Ablative fractional CO_2_ laser 125 mm handpiece, 10 mm spot, 25% density, 10 Watts	3 sessions with a 2-month interval	Complete resolution of the two facial plaques	No signs of relapse after 6 months
O’Donoghue et al. case series [29]	Lupus pernio nose; diffuse violaceous involvement and fleshy nodules on ala rim Violaceous nodule on nasal bridge, nasal swelling and erythema Coalescent fleshy violaceous nodule on nasal tip and left nasal ala	55F 57 M 58 F	Sarcoid nodules underwent debulking and recontouring with a CO_2_ laser in “paint mode” (18 W, 6 mm spot size); for cosmetic refinement, patients 1 and 3 received a resurfacing pass (14 W, 6 mm spot size), accompanied by intralesional triamcinolone acetonide (TAC) injection to prevent recurrence		New contour maintained	Six years maintained on 5 mg prednisolone, mild hypopigmentation At 14 months nasal contour was stable At 9 months, partial recurrence with nasal swelling in conjunction with flare of systemic sarcoid
Stack et al. case report [30]	Nodules on nose causing nasal obstruction	31 M	Excision with CO_2_ laser and intralesional TAC			24 months no recurrence
Piccolo et al. case report [31]	Three nodules located on the anterior and posterior aspects of the pinna	26 F	IPL 500 nm 2 pulses 5–10 ms, 12–16 J/cm^2^	4	Reduction of the vascular component and in the consistency of the lesions	Improvement persisted throughout the 6 months
Emer et al. case report [32]	Lupus pernio on the nose	Not shown	PDL 595 nm 7 mm, 0.45 ms, 8 J/cm^2^ NAFR 1550 nm (70 mJ, treatment level 6, 8 passes)	1	Reduction of lesions	N.D.
Grema et al. case report [33]	Scar sarcoid on right elbow and both knees (red-brown discoloration)	50 F	QSRL, four treatments Previously treated with PDL with no effect 585 nm, 7 mm, 0.5 ms, 5.5–5.6 J/cm^2^	4	Lightened after four treatments	No recurrence at three years
Ekbäck et al. case report [34]	Lupus pernio of cheek	57 F	PDL 585 nm, 45 ms, 6.75–7 J/cm^2^ Frequency-doubled YAG laser 532 nm, 50 ms, 12–16 J/cm^2^	10 treatments over 3 years 2 treatments over 7 months	Limited effect, less redness and thinner lesions Complete healing	N/D
Momen et al. case reports [35]	Erythematous plaque on upper lip Nodules and plaques across cheek Erythematous triangular plaque on forehead Lupus pernio	One female, age not shown Two patients, gender and age not shown Three females, age not shown One female, age not shown	Single pass of CO_2_ laser and two treatments with PDL Two treatments with CO_2_ laser Singular PDL Seven treatments with PD		Self-reported improvement of 50–60%	

PDL—Pulsed Dye Laser, IPL—Intense Pulse Light, NAFR—Nonablative Fractional Resurfacing, QSRL—Q-switched Ruby Laser.

**Table 2:** The use of lasers as a cause/progression of cutaneous sarcoidosis.

Author and Type of the Study	Clinical	Patient Age/Gender	Laser/Setting	Treatment	Outcome
Kim Hr et al. case report [36]	The enlargement of the scar	71 M	Enlargement of the scar subsequent to three sessions of PDL	Hydroxychloroquine (100 mg b.i.d.) along with topical pimecrolimus and topical steroids	Reduction of the scar
Kormeilii T et al. case report [37]	Hypertrophic scars on his left glabellar region and left side of upper lip	33 M	Progression two years after CO_2_ laser resurfacing	N/D	N/D

## Data Availability

The data presented in this study are available upon reasonable request from the corresponding author.
